# Learning from boundlessness: epistemic shifts towards a holistic worldview following psychedelic experiences

**DOI:** 10.1038/s44184-026-00186-6

**Published:** 2026-01-29

**Authors:** E. K. Argyri, F. Fraser, S. Schilling, A. Frick, O. C. Robinson, L. Roseman, C. J. A. Morgan

**Affiliations:** 1 University of Exeter, Exeter, UK; 2 University of Vienna, Vienna, Austria; 3 Regent’s University London, London, UK

**Keywords:** Human behaviour, Psychology

## Abstract

Psychedelic substances are increasingly recognized for their potential to catalyse transformative shifts in worldviews. Central to these transformations may be the integration of self-transcendent states. This mixed-methods study explored transformative psychedelic experiences, focussing on subsequent epistemic shifts. Ninety participants completed the Awe Experience Scale (AWE-S), and the Inclusion of Other in Self Scale (IOS) and open-ended questions exploring epistemic changes. The vastness and connectedness components of awe recalled from the acute psychedelic experience were significantly positively associated with perceived self–other overlap post-experience. Thematic Network Analysis revealed three epistemic shift clusters: (1) expanded awareness and openness to complexity, (2) dissolution of societal and natural boundaries, (3) increased prosociality, compassion and acceptance of difference. Together, these patterns suggest that boundary-expanding experiences can promote reflective meaning-making towards more holistic, interconnected frameworks of understanding self, others, and the world. We discuss the potential of psychedelic experiences to foster prosocial and ecologically aware worldviews, and highlight the need for further research to identify culturally attuned resources for supporting the integration of transformative psychedelic experiences.

## Introduction

Psychedelic substances have long been associated with profound transformations in consciousness, self-perception, and worldview^1–3^, with significant focus on the substances’ promising therapeutic potential for a range of mental health disorders^4,5^. In recent years, researchers have begun to unravel the mechanisms through which psychedelics catalyze such changes^6^, highlighting the essential role of the subjective effects experienced during the psychedelic ‘trip’ ^7–9^.

Mystical-type experiences, often characterized by feelings of unity, transcendence of time and space, ineffability, and deep spiritual or existential significance, have been identified as central to psychedelics’ transformative effects^10–12^. Crucially, transformative outcomes are not limited to mystical states: non-mystical experiences involving an emotional breakthrough have also been shown to predict positive therapeutic change^13^. Integral to the transformative effects of psychedelics may be their capacity to evoke awe—a self-transcendent emotion that is increasingly recognized as a key psychological mechanism underlying long-term personal growth^14,15^.

Awe is considered an *epistemic* emotion (a state where evaluations of reality are altered) arising from two appraisals: a vastness in perception (stimulus too large to comprehend with existing mental structures) and a need for cognitive accommodation (re-organisation of structures as new learning)^16^. Ihm and colleagues^17^ propose that through this need for cognitive accommodation, awe facilitates meaning making in order to make sense out of ambiguity. They further argue that awe may have played a key role in the evolution of human societies and been foundational to the emergence of religion and societal structures, while for the individual, it may be a trigger for lasting changes in worldview and identity. A frequently cited example in awe studies is the “overview effect”, reported by astronauts when viewing Earth from space^18^. The overview effect is characterized by profound awe, a collapse of perceived boundaries, and a sense of unity with humanity and the planet, often accompanied by lasting shifts in values and worldview^19^.

Accordingly, events like psychedelic, mystical or otherwise extraordinary experiences that exceed one’s bounds of interpretative capacities for making meaning require reconfiguration of one’s life story and sense of self^20^. Several studies describe transpersonal changes involving shifts in psychedelic users’ metaphysical beliefs^21–23^, yet the processes that underlie these shifts are yet to be untangled.

Predictive coding theories posit that the brain functions as a hierarchical inference machine, using top-down priors to interpret bottom-up sensory inputs^24^. Psychedelics are thought to disrupt this process, reducing the influence of high-level priors and allowing for greater input from sensory and emotional data^25^. Studies of classic psychedelics suggest shifts in salience processing, effectively lowering the threshold for significance and meaning^26–28^. In Bayesian terms, 5-HT_2A_ stimulation reduces the precision-weighting of top-down predictions, allowing a wider range of bottom-up signals to be appraised as noteworthy^25^.

This shift fosters openness to possibilities, unbound by habitual expectations, and lays the groundwork for intuitive reasoning—an embodied, rapid mode of understanding that synthesizes complex information in novel ways^29^. This freer state of cognition parallels the exploratory learning seen in children, described by Gopnik^30^ as a “high-temperature search” that prioritizes possibility over probability, exploration of the new over exploitation of the known. Psychedelics can temporarily recreate this cognitive flexibility, facilitating intuitive insights that feel revelatory and transformative^31^.

This disruption of prior frameworks for sense-making and overwhelming sense of new meaning has been termed ‘ontological shock’, a catalyst that elicits a feeling of ‘groundlessness’ within ontologically challenging psychedelic experiences and in turn necessitates changes in worldview that build new cognitive ground^32^. This process can also be understood through the lens of socio-cognitive research on Diversifying Experiences^33^. Diversifying Experiences (DEs) refer to unusual and unexpected events or situations that push individuals outside the realm of ‘normality’ and have been linked to increases in cognitive flexibility^34^. The process of reflecting on extraordinary experiences encourages a view of the world not previously considered and as such diversifies individuals’ framings of reality.

Psychedelic experiences often alter perceived boundaries, loosening distinctions between self and other, mind and body, and individual and environment. In prior research, these alterations are typically described in terms of *ego dissolution*, a diminished sense of self^35^ and *oceanic boundlessness*, a unitive state of interconnectedness^10,36^. The two phenomena are closely related and can co-occur, though they are not identical: ego dissolution highlights the attenuation of self-representation, whereas oceanic boundlessness captures the positive, unifying aspects of self-transcendence. Both psychedelic-induced ego dissolution and the feeling of oceanic boundlessness can foster a sense of interconnectedness and unity that transcends conventional separations^10,35^. Research has also indicated that psychedelic-induced ego dissolution can result in identity fusion, enhancing social bonding and prosocial behaviours^37–39^. These shifts in boundary perception may underpin changes in metaphysical beliefs and identity associated with psychedelics.

Despite growing evidence on the transformative effects of psychedelics, little is known about the kinds of epistemic shifts that underlie lasting changes in worldview and how awe experienced during psychedelic trips contributes to such changes. This study aimed to explore:

1. The kinds of epistemic changes in intuition, perceptions of normality and boundaries individuals report following transformative psychedelic experiences, alongside other shifts in their intrapersonal, interpersonal, and transpersonal understanding.
2. What aspects of the awe spectrum during a psychedelic experience relate to post-experience perceived self-other boundaries?

## Methods

### Participants and Design

This study used a mixed qualitative and quantitative cross-sectional design. Ninety participants who self-identified as having experienced long-lasting and significant changes following the use of psychedelic substances completed the online questionnaire on the survey platform Qualtrics (age range 18–75 years old, age M = 37.2, SD = 15.1). The participants were recruited via advertisements on social media and various fora including Twitter, Reddit, Facebook and Instagram. The recruitment ad text stated: “The many faces of psychedelic transformations: Have you experienced significant and long-lasting changes from psychedelics? This may include positive and/or negative significant shifts in perspective; how you perceive and/or understand yourself, others, and the world, visual or other perceptual changes”.

Inclusion criteria were (1) to identify as having experienced significant and long-lasting changes from taking a psychedelic drug (this was subjectively defined by the participant and there was no exclusion based on social context or purpose of taking the psychedelic), (2) be aged 18 or over. Participants were recruited via a range of means: The online survey was distributed via multiple social media channels and poster advertisements. There were no financial incentives for participation in the survey.

The majority (68; 75.6%) of these participants indicated living in the UK, 11 (12.2%) in other English-speaking countries (U.S.A, Canada and Australia), and 11 participants were based in non-English speaking countries across Europe and Latin America (12.2%), (with fewer than 5 participants in each country). Twenty-one participants (23.3%) resided in a country where the majority of people do not speak their native language. Demographic characteristics are presented in Table 1.

**Table 1:** Frequencies of demographic categories within the dataset

		Percentage of total sample (*n* = 90)
Gender	Female	33.3%
	Male	62.2%
	Other Gender	4.4%
Age	Aged 18-24	21.1%
	Aged 25-34	31.1%
	Aged 35-44	22.2%
	Aged 45-54	10%
	Aged 55+	15.6%
Educational level	High school educated	13.5%
	Undergraduate degree	37.8%
	Vocational or technical Qualification	2.2%
	Masters degree	30%
	PhD or other doctoral degree	13.3%
	Other/ prefer not to say	2.2%
Ethnicity	White/European	84.4%
	Latin	2.2%
	Asian	2.2%
	Black	1.1%
	Mixed Ethnicity	7.8%
	Unknown ethnicity	2.2%

### Procedure

Six pilot interviews were conducted with individuals associated with a transdisciplinary psychedelic interest group, composed of a variety of people, including academics, practitioners, and others with lived experience of psychedelic states. Through these interviews, a set of long-lasting changes from psychedelic use was identified. These included changes in how individuals understood and related to themselves, others, and the world. Previous literature on psychedelic research, along with the pilot interviews, informed the design of the survey reported in this publication and the follow-up in-depth interview study schedule.

### Data collection and ethical approval

Data were collected anonymously via an online survey created in the online survey platform Qualtrics, between December 2022 and August 2023. The questionnaire comprised a written consent form, followed by a series of open-ended and closed-ended questions. The project was approved by the University of Exeter research ethics committee.

### Quantitative measures and analyses

The Awe Experience Scale (AWE-S^40^) is a 30-item measure of awe comprising six factors: *altered time perception*, e.g., ‘I noticed time slowing’, *self-diminishment*, e.g., ‘I experienced a reduced sense of self’, *connectedness*, e.g., ‘I had the sense of being connected to everything’, *perceived vastness*, e.g., ‘I experienced something greater than myself’, *physical sensations* e.g. ‘I felt my eyes widen’ and *need for cognitive accommodation* e.g. ‘I felt challenged to understand the experience’. Participants were asked to think back to the acute effects of their most transformative psychedelic experience, under the influence of the substance, and to rate that experience in terms of agreement with each AWE-S item, based on a 7-point Likert scale ranging from Strongly disagree to Strongly agree. All subscales together had good reliability, Cronbach’s α = 0.92. Separately, the subscales also had adequate reliability: altered time-perception, Cronbach’s α = 0.91, self-diminishment, Cronbach’s α = 0.91, connectedness, Cronbach’s α = 0.94, perceived vastness, Cronbach’s α = 0.90, physical sensations, Cronbach’s α = 0.74, need for accommodation, Cronbach’s α = 0.85.

The Inclusion of Other in the Self Scale (IOS^41^) scale was a 7-point measure of interconnectedness measured through a choice of increasingly overlapping circles. We adapted this scale for the present study to measure the currently perceived degree of interconnection felt with other humans, non-human beings, and the world, respectively. The pictures were accompanied by the following instructions (taking the ‘world’ target as an example*): ‘How much overlap do you see between yourself and the rest of the world? See each statement [self–other humans/self-non-human beings/self–world] for what ‘other’ represents in the diagram and choose what’s closer to your view.’* Scores ranged from no overlap (a score of ‘0’) to almost complete overlap (a score of ‘6’). The scores for the three targets together reached adequate levels of internal consistency (Cronbach’s α = 0.85).

Pearson correlation coefficients were computed to examine the relationships between Inclusion of Other in Self (IoS) non-acute measures and Awe Scale (AWE-S) components referring to the acute experience. Although some awe subscales showed a skewed distribution, it was within the accepted tolerance of the Pearson correlation, which is robust to deviations from normality^42^. To account for the risk of Type I errors due to multiple comparisons, we applied the False Discovery Rate (FDR) correction using the Benjamini-Hochberg (B-H) procedure^43^. This method adjusts *p*-values to maintain an acceptable proportion of false discoveries among significant results, making it appropriate for exploratory analyses. All statistical analyses were conducted using SPSS (Version 29.0.1.0; IBM Corp.)

### Qualitative Method

Open-ended questions. Participants were presented with the following open-ended questions:

1. Nature of acute experience: *Reflecting on the questions above, is there anything you want to add about the nature of your acute experience (under the effects of psychedelics)?*
2. Changes in *intrapersonal* understanding: *Has your way of understanding yourself changed as part of your transformation? If yes, how?*
3. Changes in *interpersonal* understanding: *Has your way of understanding others changed as part of your transformation? If yes, how?*
4. Changes in *transpersonal* understanding: Has your way of understanding reality* changed as part of your transformation? If yes, how? *This would include any shifts in your worldview/religious/spiritual beliefs
5. Changes in perception of boundaries: *Have the boundaries you see between yourself and the rest of the world changed as a result of your transformative psychedelic experience(s)? If yes, how?*
6. Changes of intuition*: Has your intuition changed following your transformative psychedelic experience(s)? If yes, how?*
7. Changes in perception of normality: *Has your understanding of what is ‘normal’ changed following your transformative psychedelic experience(s)? If yes, how?*
8. Stability of understanding psychedelic experience: *Did your understanding of your experience remain stable, or did it change over time?*
9. Managing uncertainty: *Are you struggling more with the unexpected since your experience or feeling better equipped to manage what comes your way?*
10. Vulnerability during experience: *Did you experience increased feelings of vulnerability during your transformative experience(s) (under the direct influence of the substances)?*
11. Vulnerability after experience: *Did you experience increased feelings of vulnerability following your transformative experience(s) (not under the direct influence of the substances)?*
12. Perceived impact of sharing the experience with others: Have *you talked to others about your transformative experience(s)? If yes, how did this affect you?*

### Qualitative analysis

Structured Tabular Thematic Analysis (ST-TA) was employed to analyse the open-text data^44^. ST-TA is a form of thematic analysis designed based on both reflexive^45^ and ecumenical^46^ thematic analysis, to analyse brief texts such as written responses to open-ended survey questions. Excel spreadsheet software is used to organize the data and thematizing in ST-TA. Frequencies of themes are calculated to demonstrate how common a theme is within the dataset.

The current study was conducted using an inductive-deductive hybrid ST-TA analysis. Hybrid ST-TA analysis follows the following phases: 1. A priori theme development; 2. Deep Immersion in the Data; 3. Generating Codes and Themes; 4. Tabulating Themes Against Data Segments; 5. Checking Inter-analyst Agreement; 6. Exploring Theme Frequencies, 7. Developing thematic maps and diagrams, and 8. Producing the report^44^.

During Phase 4, the two analysts engaged in a process of establishing agreement to ensure transparency and consistency in theme formation corresponding to the data. Conclusions were reached through consensus to avoid idiosyncratic interpretations.

### Reflexivity and research process

The study was designed within a transdisciplinary research centre for psychedelic studies. During the study design phase, we used a deductive process to develop a set of questions around participants epistemic changes, based on existing literature. These formed the meta-themes that provided organising categories for data into the kinds of change reported. Within these categories, the process of deriving themes was conducted inductively, based on collating data codes into semantic groupings and then into labelled conceptual themes.

EKA and CJAM designed the survey. EKA and FF conducted the analysis of the brief written texts. The interactive dialogical approach was taken to data analysis. A series of scheduled discussions between the analysts involved intrapersonal and interpersonal reflections to inform the development of the themes.

The cyclical, iterative and inter-subjective nature of ST-TA analysis aids towards consistency and transparency. The agreement reaching process involves two analysts independently analysing part of the dataset and reaching a minimum of 80% agreement^44^. The analyst pair met to discuss theme revisions to ensure clarity and cogency until 80% inter-analyst agreement was reached. The interanalyst agreement process helps to ensure a non-solipsistic, consensual approach to analysis.

Following the first layer of inductive analysis, Thematic Network Analysis was conducted as a method of visualising the relationships between the data. EKA and AF revisited the themes and organised the data using Gephi, conducting the analysis led by SS.

Inductively generated codes were extracted from the frequency table and transformed into a co-occurrence table, consisting of which codes were coded alongside other codes (Supplementary Materials Data file). The data were imported into a social network analysis program (Gephi 0.9.5) to create a visual representation of the relationships between thematic codes and their significance within the overall network, following prior examples of network representation of qualitative data^47^.

To minimize noise and exclude co-occurrences likely to have occurred randomly, the association rule “lift” was applied as a backboning filter. The lift value was calculated as the ratio of observed co-occurrence frequency to the expected co-occurrence frequency, based on the individual occurrences of the nodes. This allowed us to quantify how much more likely two codes were to co-occur compared to random chance. To manage extreme values in the network, we capped lift values at the mean of [6.22], which represents the average co-occurrence strength. This threshold was chosen to balance the preservation of typical relationships while minimizing the impact of outliers that could disproportionately influence the network structure^47–49^. Visual exploration, using the ForceAtlas 2 algorithm, was employed to examine interactions between codes, while modularity algorithms identified codes that frequently co-occurred^50–55^. These Thematic Network Analysis graphs illustrate code relationships by representing the total occurrences of a code as node size (weighted degree), the frequency with which particular codes are discussed together in a reference as the thickness of line between codes (edge weight), the overall references shared across codes (network centrality), and clusters of closely related codes (modularity clusters). A Thematic Data Extraction Table, showing selected references, with applied codes, interpretations and the clusters they relate to, can be found in supplementary materials.

## Results

### Quantitative descriptive results

#### Psychedelic Substance Use: Age at first use, substance and number of experiences

Participants were asked how old they were at the time they first used psychedelics (age range 15–68, age M 24.8, SD 10.15). Twenty-two (24.4%) were under the age of 18, 31 (34.4%) between 19–24, 13 (14.4%) between 25–29, 7 (7.8%) between 30–34, 3 (3.3%) between 35–39, 5 (5.6%) between 40–55 and 2 (2.2%) were over 55 years old when they had their first psychedelic experience. Seven participants (7.8%) did not respond.

When asked to report the number of transformative experiences they have had, 7 participants (7.8%) only had one transformative experience. 42 (46.7%) had between 2-5 transformative experiences. 15 (16.7%) had between 6 and 10 transformative experiences and 18 (20%) had had more than 10. Eight (8.9%) participants did not respond.

Participants were asked whether they had *only* transformative experiences with psychedelics. Twenty-two (24.4%) responded yes. Fifty-nine (65.6%) responded no, and nine did not give a response. Of those that also had non-transformative psychedelic experiences, 39 (43.3%) had taken psychedelics more than 10 times, 15 (16.7%) had taken psychedelics between 6-10 times and 5 (5.6%) had taken them between 2-5 times.

Participants reported what substance they had taken during their most transformative psychedelic experiences. They could report more than one substance if appropriate. The two most commonly reported were psilocybin (47.8%, 43 participants) and LSD (41.1%, 37), followed by ayahuasca (25.6%, 23), cannabis (16.7%, 15), DMT (12.2%, 11), ketamine (10%, 9), MDMA (7.8%, 7). Other reported substances included mescaline (4.4%, 4), nitrous oxide (3.3%, 3) salvia divinorum (3.3%, 3), LSD analogues including 1P-LSD and LSA (3.3%, 3), 2 C family substances, including 2C-B and 2 Ci (3.3%, 3), 5-MEO-DMT (2.4%, 2), iboga (1.1%, 1) and amanita muscaria (1.1%, 1). Two participants (2.2%) reported using amphetamines in addition to a psychedelic.

Participants were asked how long ago they had their last transformative experience with psychedelics. Seventeen (18.9%) stated it was within the previous month, 11 (12.2%) within the previous 3 months, 6 (6.7%) within the last 6 months, 15 (16.7%) within the last year, 14 (15.6%) said it was sometime within the last 5 years, 4 (4.4%) within the last 10-20 years and 5 (5.6%) over 20 years ago.

#### Experiences following psychedelic substance use

Table 2 shows data frequencies and percentages of the sample from responses to a close-ended question on types of experiences they had following their psychedelic use. The list was informed by the types of outcomes often reported as outcomes of psychedelic experiences. Participants could select multiple responses if needed.

**Table 2:** Experiences reported following psychedelic substance use, with frequencies and percentages within the sample

Post-psychedelic experiences	Frequency	Percent
Renewed sense of understanding yourself	73	81
Renewed sense of wellbeing	68	76
Renewed sense of understanding others	67	74
Shift in metaphysical beliefs (your worldview, spiritual or religious beliefs)	65	72
Connection to something ‘more’	63	70
Renewed sense of social connectedness	60	67
Visual or sound alterations or distortions	44	49
Derealization (questioning the ‘realness’ of your reality)	43	48
Dissociation (disconnection from your normal internal or external environment)	42	47
Depersonalization (disconnection from your sense of self and personhood)	41	46
Sense of unexplained fear and/or anxiety	40	44
Paranoia (perceiving threat/negative intent, feeling others are ‘out to get you’)	32	36

Overall evaluation of psychedelic transformation Participants were asked whether they would evaluate their psychedelic transformation as ‘positive’, ‘negative’, or ‘mixed’. Fifty-eight (64.4%) responded positive, 4 (4%) negative, and 22 (24%) mixed. Six participants (6.7%) did not answer.

#### Descriptive Statistics for Awe and Inclusion of Other in Self

**Fig. 1:**
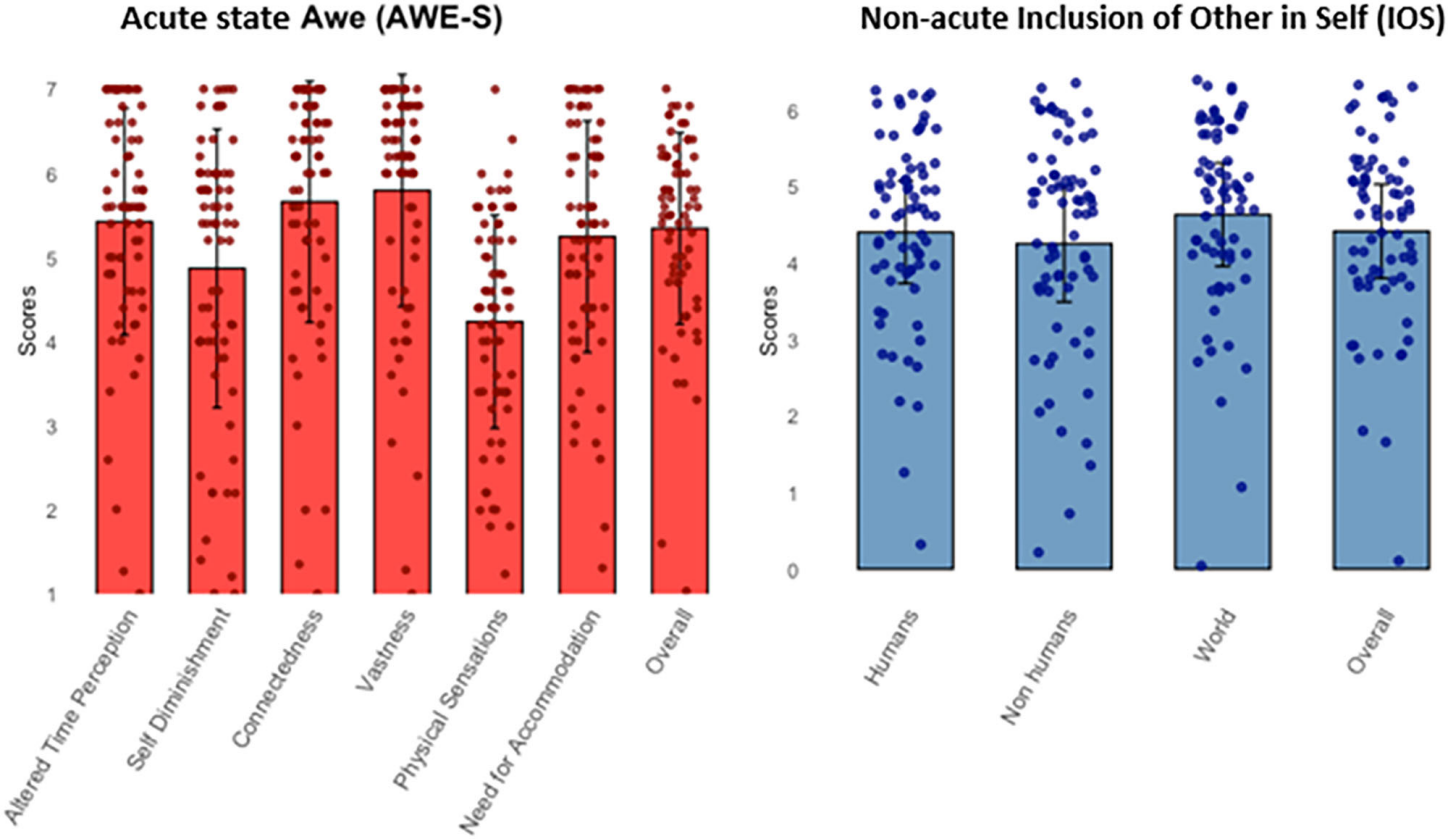
Descriptive statistics for awe and self - other measures. Means (bars), Standard Deviations (lines) and Data Distributions (dots) for AWE-S (acute) and IOS (non-acute) measures.

Descriptive statistics for IoS measures and AWE-S components are represented in Fig. 1. Participants reported moderate to high levels of overlap between the self and other (IoS measures referring to non-acute state) and substantial awe experiences (referring to acute trip state), across all AWE-S subscales. The IoS Overall measure demonstrated a mean of 4.44 (SD = 1.26), reflecting significant overlap across categories.

#### Correlation analysis between awe and inclusion of others in the self

Pearson correlation analyses revealed significant relationships between IOS (non-acute) measures and AWE-S (acute) components. To account for multiple comparisons, we applied the False Discovery Rate (FDR) correction using the Benjamini-Hochberg procedure.

**Fig. 2:**
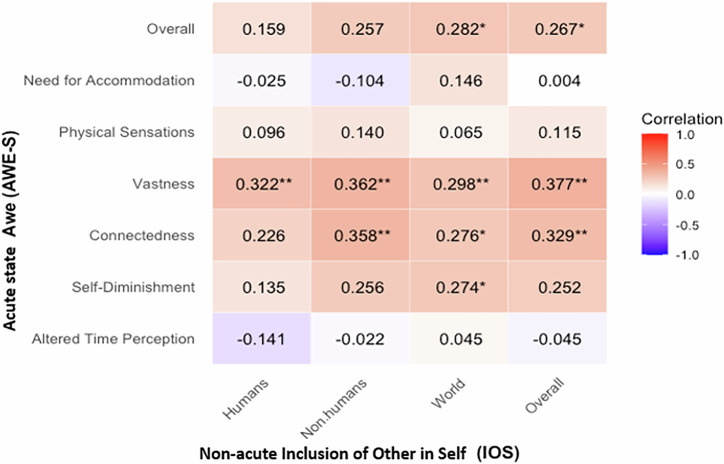
Correlation matrix for awe and self - other overlap. Pearson correlations between IoS non-acute measures and acute AWE-S components with FDR correction. Note. * = *p* ≤ .05, ** = *p* ≤ .01, FDR-corrected.

The IOS Overall (non-acute) measure was significantly positively correlated with AWE-S (acute) Connectedness (*r* = 0.329, *p*_adjusted_ = 0.002), Vastness (*r* = 0.377, *p*_adjusted_ = 0.003) and with Overall Awe (*r* = 0.267, *p*_adjusted_ = 0.047) (Fig. 2). These results indicate that higher perceptions of self-other overlap are associated with stronger awe experiences, particularly in terms of connectedness and vastness.

### Qualitative analysis of the written narratives of lasting changes

90 participants provided written accounts of the changes they experienced following their psychedelic trip.

#### Meta-theme 1: nature of acute experience

Following completion of the AWE-S scale retrospectively referring to their trip, participants were asked to share additional reflections on their acute psychedelic experience. Sixty-seven provided responses, with ten themes emerging. The most common, *Emergence of Insights and Purpose* (23%), involved increased clarity and direction. *Sense of Oneness and Connectedness* (20%) described a profound unity with everything, while *Suffering, Death, and Challenging Experiences* (16%) captured distressing or overwhelming effects.

Other themes reflected encounters with something beyond the ordinary. *Experience of Something Greater Than Oneself* (13%) described perceived guidance from an external wisdom, while *Positive Emotions* (12%) included feelings of love, bliss, and awe. *Sense of Profundity or Higher Reality* (10%) involved the perception of hidden aspects of reality, and *Religious or Spiritual Experience* (8%) reflected newfound spiritual awareness. Additional themes included *Themes of Nature* (7%), *Expansion and Wholeness of Self* (7%), and *Questioning the Nature of Reality* (7%), reflecting altered perceptions of self, nature, and existence. Example quotes are presented in Supplementary Table 1.

### Post-experience epistemic changes

#### Meta-theme 2; Intrapersonal changes: changes to understanding of self

Seventy-nine participants (88%) reported changes in their understanding of the self, reflecting shifts in self-awareness, values, and relational perspectives. Thematic analysis identified eight interrelated themes; These include *Greater Self-Insight* (42%), characterized by increased self-awareness and recognition of personal patterns; *Increased Compassion* (24%), marked by greater kindness toward oneself and others; and a *Shift in Purpose and Values* (23%), reflecting re-evaluations of priorities and moral orientations. Some described an *Increased Awareness of Wholeness and Complexity* (17%), seeing themselves as multifaceted and shaped by conditioning, while others reported *Increased Authenticity* (11%), feeling more aligned with their true desires. Some reported general improvements in *Wellbeing* (8%), whereas *Increased Awareness of Oneness* (8%) reflected a dissolution of self-boundaries. A minority (3%) reported significant *Negative Repercussions of Change*, struggling with distressing insights. Example quotes are presented in Supplementary Table 2.

#### Meta-Theme 3: interpersonal changes: Changes to understanding of others

Seventy-three participants (81%) reported changes in their understanding of others, reflecting shifts in empathy, perspective-taking, and relational awareness. Thematic analysis identified six interrelated themes. The most common, *Increased Empathy* (28%), involved a heightened ability to understand and forgive others, often accompanied by *Increased Acceptance of Difference* (24%), where participants recognized that individuals construct and interpret the world in unique ways. *Awareness of Commonality in Human Experience* (23%) emerged as participants acknowledged shared struggles and emotions, while *Interrelatedness* (21%) reflected a broader recognition of interconnectedness and the relations between parts, shifting from an “us vs. them” mentality to a more unified perspective. *Increased Compassion* (18%) was reported as a deepened sense of kindness and consideration for others, while *Sense of Sonder* (10%) captured the realization that others are complex, whole individuals with their own inner worlds. Example quotes are presented in Supplementary Table 3.

#### Meta-Theme 4: transpersonal changes: changes to understanding of reality

Seventy-four participants (82%) reported changes in their understanding of reality, reflecting shifts in metaphysical, existential, and epistemological perspectives. Thematic analysis identified ten interrelated themes. The most common, *Increased Sense of Animism* (29%), involved perceiving nature as more alive and communicative. *Increased Awareness of the Subjectivity of Reality* (18%) followed, with participants recognizing reality as perspectival rather than absolute. Some experienced a *Shift in Life Purpose and Values* (11%), while others described a heightened sense of *Interrelatedness* (9%), perceiving deeper connections across systems and existence. Several themes reflected changes in belief frameworks. *Reductionism Reduction* (9%) marked a move away from strictly physicalist perspectives, while *Increased Religiosity* (9%) indicated shifts toward faith or spiritual conviction. Some reported an *Increase in Philosophical Inquiry* (9%), engaging more deeply with existential and metaphysical questions, or *Increased Openness and Curiosity* (8%), returning to a more exploratory, childlike perspective. *Perception of Mortality* (7%) often shifted toward reduced fear of death, while *Increased Agnosticism* (6%) reflected greater uncertainty about prior beliefs. A small minority (2%) reported significant *Negative Change*, describing distressing and destabilizing shifts in worldview. Example quotes are presented in Supplementary Table 4.

#### Meta-theme 5: Changes to understanding of ‘normal’

Seventy-one participants (79%) reported changes in their understanding of ‘normal,’ reflecting shifts in its boundaries, origins, and significance. Thematic analysis identified nine interrelated themes. The most common, *Greater Acceptance of Difference and Other Perspectives* (22%), involved recognizing that what is considered normal varies across individuals and contexts. Seeing *Normality as a Social Construct* (21%) emphasized that norms are shaped by cultural and societal conditioning rather than inherent truths. Some described *an Expanded Concept of Normality* (20%), broadening their sense of what is possible and acceptable.

Other themes reflected deeper ontological and epistemic shifts. *Complexity Awareness* (17%) involved a recognition of the fluid and multifaceted nature of human perception, while *Metaphysical Resonance* (11%) captured an increased sense of sacredness or divine reality. Some participants described *Rejecting Normal* (8%), rejecting the concept entirely as a limiting construct, while others reported a strengthened *Nature Connection* (7%) and *Interrelatedness* (4%), seeing normality in terms of interconnected systems rather than fixed societal expectations. A small minority (2%) reported *Negative Changes*, describing paranoia and heightened fear responses. Example quotes are presented in Supplementary Table 5.

#### Meta-theme 6: Changes in intuition

Sixty-eight participants (76%) reported changes in their intuition, reflecting shifts in how they experience, trust, and apply it. Thematic analysis identified seven interrelated themes. The most common, *Greater Guidance and Attunement to Intuition* (21%), involved participants relying more on intuition to navigate decisions and feeling a stronger connection to their inner sense of truth. *Greater Trust and Faith in Intuition* (18%) reflected an increased willingness to trust intuition as part of a broader sense of surrender to life’s unfolding. *Shift in Purpose and Values* (17%) captured how intuitive insights led participants to reassess priorities, often letting go of material concerns or control over external circumstances. Other themes suggested a widening of intuitive perception. *Interrelatedness* (14%) appeared in changes to relationships, particularly in family dynamics, where intuition fostered a sense of gratitude and reciprocity. *Metaphysical Resonance* (13%) reflected an intuitive openness to spirituality, sacredness, or the presence of a divine force. *Openness* (10%) described feeling more receptive and mindful, while *Greater Alignment with Self* (9%) involved deeper bodily and emotional awareness. Additional themes included *Complexity Awareness* (8%), *Nature Connection* (7%), and *Empathy* (6%), while a small minority (4%) reported *Negative Change*, describing distressing or disorienting shifts. Example quotes are presented in Supplementary Table 6.

#### Meta-theme 7: Changes of boundaries between self and world

Seventy-two participants (80%) reported changes in their perception of boundaries between their self and the world following their transformative psychedelic experiences. Thematic analysis identified six themes. The most common, *Interrelatedness* (30%), reflected a heightened sense of interconnection, with participants describing understanding everything plays a role and has a place in a greater whole. *Malleability* (12%) captured perceptions of self-world boundaries as fluid and changeable. Some participants dismissed *Boundaries as Illusory* (11%), expressing the view that self-world divisions are temporary perceptions rather than inherent realities. *Boundlessness* (10%) described the removal of borders and boundaries in their perception, while *Oneness* (10%) reflected a more nuanced sense of unity, where participants expressed awareness that they are metaphysically indistinct from their surroundings. A minority (2%) reported Increased Disconnection, describing a shift in the opposite direction where their experiences led to feelings of isolation or detachment. Example quotes are presented in Supplementary Table 7.

#### Meta-theme 8: Vulnerability during the experience

Sixty-four (71%) participants experienced increased feelings of vulnerability during their transformative psychedelic experience. Sixteen (18%) experienced no increase and 10 did not respond. Seventeen (19%) participants did not specify the valence of this increase, 8 (9%) reported a positive experience of vulnerability, 9 (10%) reported both positive and negative experience of vulnerability, while 17 (19%) reported a negative experience of vulnerability during their transformative trip (e.g. *Yes, I was very confused and scared at times. Had a difficult time grasping what was going on and felt that the world was a scary place*).

#### Meta-theme 9: Post-experience vulnerability

Forty (44%) participants experienced increased feelings of vulnerability following their transformative psychedelic experience. Eight (9%) reported *positive outcomes of vulnerability*, 9 (10%) reported a *mix of positive and negative outcomes*, while 11 (12%) reported significant *negative effects of* their *increased vulnerability* (e.g. *Yes, I lost complete sense of self and had trouble talking to even my closest friends*). Fourteen (16%) did not specify the valence of this increase.

#### Meta-theme 10: Managing uncertainty

Participants were asked whether they struggled more or felt better equipped to manage the unexpected, following their transformative experience. Fifty-one participants (57%) felt *better equipped to manage the unexpected*, while 3 (3%) were *struggling more*. Fifteen (17%) responded that they *struggled more in some cases and felt better equipped in others*.

#### Meta-theme 11: Stability of understanding the experience

Participants were asked whether their understanding of their experience remained stable over time. 23 (26%) participants reported their understanding *remained stable*, 49 (54%) reported their *understanding changed with time*, while 11 (12%) of participants identified both changing and stable parts within their understanding.

#### Meta-theme 12: Sharing the experience with others

Participants were asked whether they talked to others about their experience and how this affected them. For 36 (40%) participants, *sharing their experienced with others helped* them, 13 (14%) experienced *mixed effects of sharing*, while 4 (4%) found *sharing hurt* them in some way (e.g. *Yes, my family, friends and work colleagues, this resulted in being fired from work*). Twenty-one (23%) did not specify how their sharing affected them.

### Thematic network analysis

The 34 themes relating to post-experience epistemic shifts that were identified through the Structured Tabular Thematic Analysis were included in the thematic network analysis graph. Three overarching clusters of epistemic shift themes were identified through the Thematic Network Analysis.

**Fig. 3:**
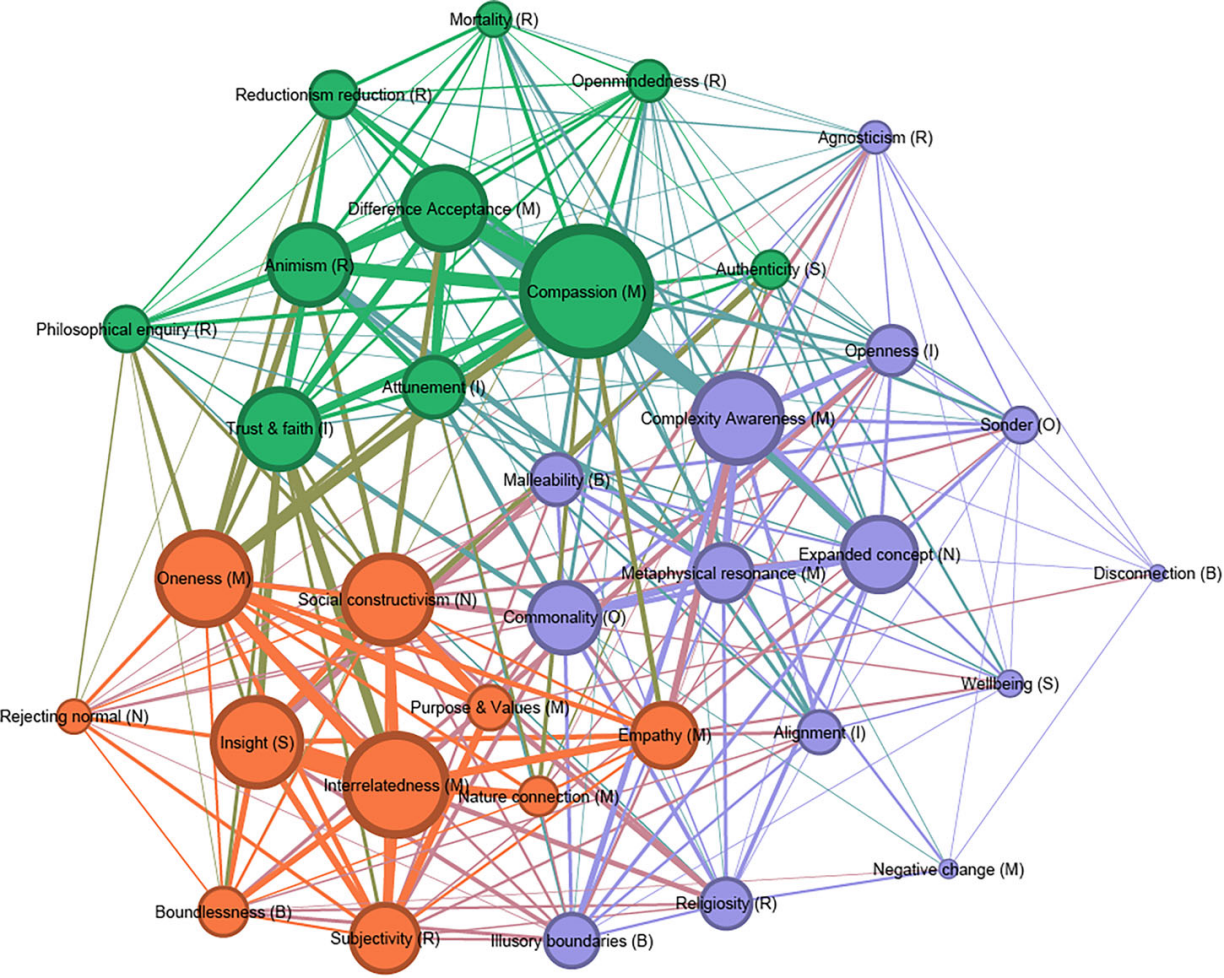
Thematic network of post-psychedelic epistemic shifts. Node size represents theme frequency (weighted degree); line thickness indicates co-occurrence frequency (edge weight). Abbreviations in brackets denote origin question: S= Understanding of self; O= Understanding of others; R=Understanding of reality; I= Changes in intuition; N= Understanding of normal; B= Understanding of boundaries of self and world; M= themes occurring across multiple questions.

Figure 3 shows the thematic network with the 34 themes, illustrating the overarching clusters and their connections: Dissolution of societal and natural boundaries (orange), Expanded awareness and openness to the complexity of reality (purple), Increased prosociality, compassion and acceptance of difference (green).

The 14 themes within this thematic cluster (Table 3) reflect participants’ heightened awareness of the complexity of reality, paired with an increased openness to uncertainty and ambiguity, and to the supernatural.

**Table 3:** Thematic cluster 1: Expanded awareness and openness to the complexity of reality

**Expanded Awareness and Openness to the Complexity of Reality (representing 41.18% of Graph)**	Complexity Awareness (M)	*Awareness of the complexity of reality and people, their different parts and how they form a bigger picture*
	Expanded concept (N)	*Expanded concept of what is normal*
	Commonality (O)	*Recognising the commonality in human experience and suffering*
	Metaphysical resonance (M)	*Increased resonance to metaphysical ideas and experiences*
	Illusory boundaries (B)	*Awareness of illusory nature of boundaries*
	Malleability (B)	*Increased sense of malleability of reality’s boundaries*
	Religiosity (R)	*Increased religiosity*
	Openness (I)	*Increased openness*
	Alignment (I)	*Increased alignment to Self*
	Sonder (O)	*Recognizing and appreciating the wholeness, depth and complexity of others’ lives*
	Agnosticism (R)	*Increased agnosticism and uncertainty*
	Wellbeing (S)	*Increased wellbeing*
	Negative change (M)	*Negative changes*
	Disconnection (B)	*Increased disconnection and isolation*

Participants frequently described that their understanding of what is normal had expanded, which correlated with their increased awareness of the complexity of reality, intuitive openness and resonating with metaphysical ideas and experiences such as supernatural phenomena. This resonance, in turn, cooccurred with participants’ increased alignment with their intuition, an awareness of the malleable nature of reality’s boundaries and increased awareness of the commonality in human experiences. Descriptions of intuitive openness also cooccurred with agnosticism, a sense of uncertainty about the nature of reality. Sonder (an appreciation of others as having fully fledged independent realities), had some connections with agnosticism but also to increased wellbeing and feelings of disconnection and other negative changes. Negative change had some cooccurrences with religiosity, which in turn related to metaphysical resonance, an expanded concept of normal, and a sense of the illusory nature of reality’s boundaries.

The 10 themes in this cluster (Table 4) primarily capture participants’ experiences of profound shifts in their perception of boundaries—both internal and external. These shifts point to a reconfiguration of the perceived separation between self and world.

**Table 4:** Thematic cluster 2: Dissolution of societal and natural boundaries

**Dissolution of societal and natural boundaries (representing 29.41% of Graph)**	Interrelatedness (M)	*Awareness of interconnectedness and interdependence*
	Oneness (M)	*Awareness of oneness, unity of all things*
	Insight (S)	*Emergence of insight*
	Social constructivism (N)	*Awareness of the socially constructed nature of normality*
	Subjectivity (R)	*Increased awareness of the subjectivity of reality*
	Empathy (M)	*Increased empathy and emotional understanding*
	Boundlessness (B)	*Dissolution of boundaries*
	Purpose & Values (M)	*Redefined purpose and values*
	Nature connection (M)	*Increased awareness and connection to the natural world*
	Rejecting normal (N)	*Rejecting the concept of normality*

Participants frequently described that their experiences led them to significant insight about themselves, which coincided with an increased awareness of the socially constructed nature of reality and which, in turn, along with oneness and an appreciation of the subjectivity and many perspectives on reality, strongly related to a newfound purpose and redefined values in life.

Participants’ new insight about themselves most frequently cooccurred with their understanding of interrelatedness, oneness, and boundlessness, where they reflected on their unity with the rest of the world and the relationships between them and other parts of an ‘interconnected web of reality’. This interrelatedness also frequently co-occurred with an understanding of the subjectivity of reality, which co-occurred with the rejection of the concept of normal and empathy, an increased emotional understanding of others and their perspective, and with increased connection to the natural environment.

**Table 5:** Thematic Cluster 3: Increased prosociality, compassion and acceptance of difference

**Increased prosociality, compassion and acceptance of difference (representing 29.41% of Graph)**	Compassion (M)	*Compassion and love*
	Difference Acceptance (M)	*Acceptance of different perspectives*
	Animism (R)	*Increased perception of animism, spirit and sacredness in the world*
	Trust & faith (I)	*Trust and faith in intuition*
	Attunement (I)	*Increased attunement to intuition*
	Reductionism reduction (R)	*A shift away from reductionism and physicalist worldviews*
	Philosophical enquiry (R)	*Increased engagement with philosophical ideas*
	Openmindedness (R)	*Increased openmindedness*
	Authenticity (S)	*Increased authenticity*
	Mortality (R)	*A shift in perception of death*

The 10 themes within this cluster (Table 5) highlight participants’ transformations in their relationships with others and their worldview. These changes were marked by an increased capacity for compassion, acceptance of difference, trust and faith in intuitive guidance, and a deepened philosophical and existential curiosity.

Participants frequently described heightened feelings of compassion, both towards themselves and others. These descriptions of compassion are strongly related to the second cluster’s theme of complexity awareness, also reflected in the high betweenness centrality of the thematic code. Compassion descriptions cooccurred in high frequency with an acceptance of difference and different perspectives, an increased perception of animism, spirits and entities, attunement to, and trust and faith in intuition, and a reduction in reductionist lenses for understanding reality.

Increases in compassion were also strongly linked to the second cluster’s theme of oneness and cooccurred, albeit less frequently, with increased authenticity, open-mindedness, a shift to a more accepting perception of mortality and increased philosophical enquiry.

## Discussion

This study explored the nature of transformative psychedelic experiences and subsequent changes in sense-making. In our exploratory correlational analysis, retrospectively reported awe—particularly its aspects of vastness and connectedness—was related to perceived overlap between self and other. Following quantitative and qualitative analyses of acute and lasting changes in understanding, a Thematic Network Analysis identified three interrelated clusters of epistemic shifts: Dissolution of societal and natural boundaries, Expanded awareness and openness to complexity, and Increased prosociality, compassion, and acceptance of difference.

Beyond high scores of awe, when reflecting on their transformative psychedelic experiences, participants commonly described the emergence of insight and purpose. Prior research suggests that such feelings of insight may mediate the benefits of psychedelic experiences by changing the way individuals relate to themselves and the world (their fundamental understandings)^56,57^. While these insights can catalyse lasting change, they may also risk generating over-confidence or false conclusions if not critically integrated^57^.

Awe, particularly its connectedness and vastness aspects, was positively associated with perceived self–other overlap, suggesting that boundary-expanding emotions may play a role in how individuals integrate psychedelic experiences. Song et al. ^58^ identify ‘vastness vis-à-vis the self’, a reorientation towards seeing oneself as part of a greater whole, as the core pathway linking awe to feelings of unity and mutual strengthening. This aligns with prior work connecting awe to transcendence and reduced maladaptive narcissism through heightened interconnectedness^14,15,59^. The vastness of awe can be understood as an encounter with stimuli larger than the self that overwhelms prior cognitive frames about the boundaries of self and world, and prompts reflection on one’s relation to a wider whole^60^. In our sample, over 40% explicitly described such oneness and interconnection, echoing Chen and Mongrain’s^61^ account of awe as a self-expansive emotion that deepens connection with humanity and the natural world.

Awe experiences can be situated within the broader ecology of *holotropic* (from *holos*, whole, and trepein, moving toward) experiences, a term used to describe a wide class of non-ordinary states, including those induced by psychedelics, that orient consciousness toward integration and wholeness^62^. Within this framework, awe, emotional breakthroughs, communitas, can be understood as interrelated varieties of holotropic states that expand boundaries and prompt the accommodative updating of beliefs. Recent integrative accounts likewise suggest that such phenomena converge on shared psychological and neurobiological mechanisms, including 5-HT_2A_ receptor agonism and the relaxation of high-level priors^6^. Drawing on the notion of *emergent phenomenology* proposed by Sandilands and Ingram^63^ to describe experiences that arise from complex systems in ways that cannot be predicted from their constituent parts, these transformations can be understood as emergent processes through which boundary looseing is interatively integrated, allowing new coherence of meaning and identity to develop. Related literature on *wonder*^64^ highlights how reflective, boundary-expanding emotions orient individuals toward mystery and pluralism, suggesting that epistemic transformation likely arises not directly from the acute state itself, but from its reflective integration.

The first epistemic cluster captured increased awareness of the complexity of reality and humans, an expanded sense of perspective, and increased openness to uncertainty and the unknown. Others have argued for the value of this expanded view of a ‘bigger picture’ during psychedelic experience, as an insight with profound effects for individuals’ later perspective-taking ability^65,66^ and potential for knock-on impact on wider social change^67^. However, individuals’ pre-existing belief systems, religious orientations, or contemplative practices likely shape both the appraisal and subsequent integration of these experiences.

Relatedly, over half of the participants reported feeling better equipped to manage the unexpected following their experience. The ability to navigate epistemic disruption and reconstruct meaning may depend on how resourced individuals feel to manage uncertainty, with implications for both psychological integration and distress^32,68^. Prior frameworks provide interpretive scaffolds that can either buffer ontological disruption by offering meaningful metaphors and rituals, or, conversely, intensify conflict when the experience challenges doctrinal beliefs^17,69,70^.

The second thematic cluster emphasizes shifts in participants’ understanding of interconnectedness, subjectivity, and the socially constructed nature of norms. Psychedelic experiences often involve the dissolution of boundaries between self and other, individual and environment, and societal constructs^35,71,72^. Participants described significant transformations in how they relate to the world and its structures. Key themes of insight, social constructivism, and awareness of the subjectivity of reality reflected realizations that norms and realities are co-created through shared human beliefs and interactions rather than being fixed or objective^73^. Such insights appear to dissolve the ‘taken-for-granted’ quality of social norms and boundaries, revealing them as contingent narratives rather than fixed realities and enabling a more reflective stance toward the frameworks shaping one’s worldview. This cluster also reflects an expansion of selfhood in relation to others and the world, suggesting epistemic shifts that accommodate the loosening of perceptual and conceptual boundaries that is characteristic of psychedelic experiences.

The final cluster highlights participants’ deepened compassion, acceptance of diverse perspectives, and trust in intuitive and spiritual dimensions. These shifts suggest more inclusive and prosocial attitudes grounded in greater empathy and care for others, aligning with research linking psychedelic experiences to prosocial behaviour and enhanced empathy through reduced self–other boundaries^37,74,75^. Participants’ recognition of multiple perspectives and reduced reductionist thinking indicates a more integrative approach to reality, which can hold scientific, philosophical, and spiritual framings in dialogue. The embrace of complexity and uncertainty has been associated with greater tolerance of ambiguity and enhanced interpersonal understanding^76^, alongside a re-alignment with personal values and enhanced authenticity in relationships^77^. The cluster’s themes also connect to moral expansiveness, which describes the widening of one’s moral circle to include other humans, non-humans, and the environment^78^. Participants’ reports of animistic perspectives, connection to nature, and reduced fear of mortality mirror findings from psychedelic research emphasizing enhanced ecological awareness and concern for the environment^79–81^.

Our findings demonstrate that transformative psychedelic experiences can catalyze profound epistemic shifts that may tend towards a holistic worldview. When ontological disruption is constructively integrated^32^, transformative trips can act as *diversifying experiences*^34^ that expand conceptual boundaries and promote psychological growth. Increases in compassion, emotional understanding and tolerance for uncertainty suggest psychological growth towards prosociality and align with models of transformative learning, where disorienting dilemmas prompt reflection and perspective change^32,73,82–84^. This disruption of taken-for-granted assumptions can foster a reflective stance towards societal norms and collective belief systems, encouraging more open and critical perspectives. Perlin and Li^85^ proposed that awe’s accommodative processing – the reflective integration of events that challenge prior frames of reference- can foster a ‘quiet ego’, a mature self-concept characterized by appreciation of self–other interdependence^86^ (see also Loevinger^87^). The integration of boundary-challenging insights can thus support reconfiguration of values and priorities, enhancing relational and emotional maturity.

These patterns align with arguments by Mollaahmetoglu and colleagues^66^ and Alexander Beiner^67^ that highlight the potential of such insights for individual and collective transformation. They also corroborate prior studies linking psychedelic experiences to ecological awareness^79–81^, suggesting an expanded moral and perceptual framework that encompasses a broader understanding of interconnectedness and interdependence^61^. Together, these findings indicate that psychedelic experiences can provide fertile ground for cultivating a holistic worldview, one characterised by openness, complexity, and prosocial orientation through epistemic transformation.

This study has several limitations. Participants were self-selected and predominantly evaluated their transformations positively, potentially reflecting advocacy bias. Those strongly identifying with psychedelic culture may have framed their experiences through shared cultural narratives (e.g., media or online discourse). Future studies could balance sampling by recruiting through harm-reduction or integration networks and include measures of psychedelic advocacy or cultural immersion as covariates to assess bias effects.

In addition, the quantitative results showing correlations between AWE-S connectedness and vastness and non-acute IOS scores ought to be interpreted with caution. Due to the cross-sectional, retrospective design of our study, and the debated construct validity and dimensional specificity of the AWE-S and its subscales, it’s not possible to infer causal relationships from our analyses. Future longitudinal or experimental studies (e.g., pre-/post-session awe measures and IOS with ecological momentary assessment of awe) are needed to clarify these relationships and mechanisms.

Although most participants described positive outcomes, many also reported challenging content. Acutely challenging experiences, colloquially known as ‘bad trips’ are often related to, but distinct from extended difficulties, such as those reported in Evans and colleagues’ study, based on whether the distress persists^88^. Roughly half experienced perceptual or dissociative effects following their experience and 35–45% reported anxiety or paranoia, rates higher than in controlled trials^89,90^. A few described significant longer-term distress, emphasising the need for targeted harm-reduction and post-experience support (see negative experiences section C in supplementary materials for further details). Future research should include balanced samples of positive and difficult transformations to examine differential meaning-making and adaptive resources.

Because the time since experience varied widely, recall and interpretation likely evolved differently over time, as we see suggested in our data (more than half of participants indicated their understanding has been a continuously evolving process); future work could explore the differences in narrative form, structure and content across levels of duration since the psychedelic experience within longitudinal studies. Although our data supports links between psychedelic experiences, compassion, and awareness of interrelatedness, whether such changes translate into sustained behaviour remains unclear. As Evans^91^ notes, spiritual bypassing or feelings of superiority can undermine genuine empathy, underscoring the need for cautious interpretation to avoid reinforcing psychedelic exceptionalism. The findings of this study need to be interpreted with caution in order not to further contribute to the harmful narratives of psychedelic hype and evangelism^88,92,93^.

Finally, both participants and researchers of this study were largely situated within WEIRD (Western, Educated, Industrialised, Rich, Democratic) cultural contexts, which can shape assumptions about selfhood and boundary dissolution^70^. For example, individuals embedded within cultures with higher ‘porosity’ (belief in more permeable boundaries between self and world) may be more likely to normalize spiritual or absorptive experiences as inherent aspects of reality and approach boundlessness as a constant, underlying reality^69^. Cross-cultural studies examining independent/interdependent models of self and culturally embedded metaphors of boundlessness could illuminate how meaning-making frameworks mediate both benefit and risk, informing more context-sensitive integration and harm-reduction practices.

Our findings bear practical implications for integration support and harm reduction. Because boundary loosening can be disorienting as well as beneficial, psychedelic integration may benefit from including *boundary-work*: helping individuals name, normalise, and titrate shifts in self–other and person–world distinctions, and differentiate adaptive insight from overgeneralised metaphysical conclusions or spiritual bypassing (e.g., superiority narratives)^77,85,91^. Group-based integration can leverage *communitas* to consolidate prosocial shifts while providing peer support and social normalisation for ontological disruption^32,37,39^. Harm-reduction approaches can anticipate transient derealisation, depersonalisation, or anxiety, offer concrete coping plans (e.g., sleep, nutrition, social connection) and schedule proactive check-ins during the sub-acute window^88,94^. Finally, given links between awe and moral expansiveness, integration could invite values-consistent, low-stakes prosocial or ecological actions to translate felt interconnectedness into grounded behaviour^74,78,79^.

This study explored the acute experiences and epistemic changes reported by individuals who had undergone transformative psychedelic experiences. Experiences of high awe, particularly its dimensions of vastness and connectedness, were associated with high perceived self–other overlap, mirroring the boundary loosening also evident in participants’ narratives. Such alterations in self-boundary perception may underpin broader epistemic shifts: an expanded awareness of a ‘bigger picture,’ reconfigured conceptual boundaries, and greater acceptance of difference. Many participants described increased awareness of complexity, increased compassion and a renewed alignment with their intuition, values and purpose, alongside openness to multiple perspectives. Together, these patterns reflect the complex meaning-making processes psychedelics can catalyse, aligning with transformative learning theories that frame perspective change as emerging through the reflective integration of novel insights.

For many, integration involved ongoing alignment with intuition, values, and purpose, while some faced lasting confusion and distress. These results highlight that epistemic change can be both generative and destabilising. Adaptive integration likely depends on available resources for uncertainty management and supportive frameworks for meaning reconstruction. Further research is needed to examine cultural and temporal factors shaping how boundary-challenging insights translate into enduring worldview and behavioural change.

## Supplementary information

Supplementary Materials Tables

Supplementary Materials Data

## Data Availability

Due to the sensitive and potentially identifiable nature of the qualitative data collected, and in accordance with ethical approval conditions, the datasets generated and analysed during this study are not publicly available. De-identified data supporting the findings of this study are provided within the manuscript and its Supplementary Materials.
