# Supplementary material for: Learning from boundlessness: epistemic shifts towards a holistic worldview following psychedelic experiences
Source: Npj Ment Health Res. 2026 Jan 29;5:6. doi: 10.1038/s44184-026-00186-6 (PMC12855808; doi:10.1038/s44184-026-00186-6)
Supplement: Supplementary file 1 — Supplementary Materials Tables [file 44184_2026_186_MOESM1_ESM.pdf]

Supplementary Materials for 'Learning from Boundlessness: Epistemic shifts towards a holistic worldview following psychedelic experiences'

A. Thematic Data Extraction Table with selected references, applied codes, interpretations, and community

Thematic Data Extraction Example Table			
**Example Reference**	**Thematic Codes applied to reference**	**Interpretation of Reference**	**Thematic Clusters derived through modularity algorithm***
My love for the planet, and nature has increased; I have better and more genuine relationships with people and aim to be my best self. It also made me better at comprehending complex ideas, I think it genuinely made me develop a more nuanced understanding of how abstract ideas like economics and people work as I was thinking more deeply about them in a less biased way. Generally I feel more indifferent to everything in a stoic and positive way, not a depressed way; I understand the power of acceptance and although life is tough sometimes I remain happy because I understand it comes within.	Complexity Awareness, Openness, Nature Connection, Interrelatedness	An improved understanding and relating with the rest of the world allowed for a more stoic appreciation of the complexity of reality and its parts.	**Expanded Awareness and Openness to the Complexity of Reality (representing 41.18% of Graph)**
I'm so many ways that I would be impossible to put into words fully - but the obvious one is a relinquishing if the idea what we can / should fully understand all aspects of the world and nature.	Complexity awareness	An awareness and acceptance of the complexity of reality and limitations of human thought.	
My views on normality vs deviance, sickness vs health, truth, delusion, religion and so on were radically changed. I came away with a far greater appreciation of the vastness of the landscape of human experience, and the extraordinary extent to which we each inhabit our own constructed worlds based on the input to and structure of our conscious experience.	Expanded concept of normality, Complexity awareness, Difference acceptance, Social constructivism	An expansion of concepts alongside an appreciation of the vastness of possibility within conscious experience.	

I like to think that my understanding of other people's insecurities and worries improved because I saw that we were all so similar and if I am having that thought then of course that person could be too.	Commonality, Empathy, Difference acceptance	An awareness of commonality between people led to acceptance of differences and perceived flaws, empathetic understanding.	
My beliefs have changed. I now trust in mystical experiences and believe that they are helpful. I do not believe that there are any limits to reality.	Metaphysical resonance, Expanded concept of normality	A resonance with metaphysical experiences relates to an expansion of the perceived limits of what's expected in reality	
I believe that the boundaries we see between ourselves and the rest of the world are a temporary illusion.	Illusory boundaries	An appraisal of boundaries between oneself and others as illusory.	
I believe that my self is an illusion and that there is no difference between myself and others.	Illusory boundaries, Interrelatedness	An appraisal of boundaries between oneself and others as illusory connects to an understanding of interrelatedness.	
More porous: spooky actions at distances (thinking about someone then them calling you), synchronicities (bumping into the right people at the right time or seeing lessons in the everyday) increase in quantity and quality after good experiences. Concomitantly, they decrease in quantity and quality after bad experiences.	Malleability of boundaries	A belief that the boundaries of reality are malleable and affected by the quality of psychedelic experiences.	
I became a Christian and now have a strong every day awareness of Jesus presence and guidance in my life :) But I should clarify that the Cannabis / LSD was not directly a help in coming to this transformation. It actually led me into a disastrous psychotic meltdown, not helped in any way by the adoption of eastern / new age / shamanistic belief systems that dissolved my hold on reality. When Jesus broke in it (seeming like bad news initially) was a rescue operation. And without Him I think I'd be in schizophrenic wasteland, probably still taking the drugs that got me into it.	Religiosity, Negative change	A shift to religiosity and a recognition that psychedelic experiences led to negative changes, such as psychosis.	
I was much more open at first. I experienced a messianic shift for months after the primary experience leading to large amounts of magical thinking and proselytization of psychedelics. I believed my "inner knowing" was connected to something much larger than myself, and used the intuitive "knowing" to inform some major decisions. While I do not regret those decisions, I can safely say I	Openness, metaphysical resonance	An increase in openness and a resonance to metaphysical ideas and experiences such as being guided through intuition by something greater.	

have a more grounded approach at this time in life.			
I am less inclined to do things that do not ring true to who I am. I am less compliant with outside demands, my intuition guides me through all decisions that I make. I connect to myself before taking any decisions and i would call that intuition.	Alignment to self, Attunement to intuition	An alignment to what feels true to the self and an attunement to intuition for decision making.	
Learning to adjust my own mapping of the world allowed me to understand that everyone has their own mapping and structures their own categories of evaluation often entirely different to my own.	Sonder, Acceptance of difference	A recognition of others as independent entities with their own realities and an acceptance of their differences.	
I'm more uncertain than I have ever been about any of my beliefs.	Agnosticism	An increase in uncertainty about own beliefs.	
When I find myself stressed or anxious about general life life worries I try to think back on the feeling that life isn't all that important and that we should make the most of it (as in my experience a higher being was explaining this to me).	Wellbeing, Purpose and Values shift	An improvement in emotional wellbeing accompanied by a shift in values and priorities in life.	
I feel more detached and a lessened sense of belonging	Disconnection	An increase in detachment and disconnection.	
I have since been left in a state of psychosis, and have had my life ruined because of this substance, I wish I had never touched them	Negative change	Negative changes including psychosis and regret for having one's life 'ruined' as a result of psychedelic experiences.	
I can now see much more clearly that all life of Earth is connected because it all comes from the same origin. Every iteration of biological life from the beginning is the continual dividing and replication of the original cell. Although selfhood is necessary to function as an independent organism, I can see that selves are essentially masks through which we experience the world and one another. Underneath it, we are essentially all the same 'stuff', the same facet of nature experiencing itself.	Interrelatedness, Commonality	An understanding of interrelatedness through awareness of the interconnected nature between life forms and their commonality	**Dissolution of societal and natural boundaries (representing 29.41% of Graph)**

I believe that the world is me and I am the world , I am only experiencing reality from an individual perspective because that is the way that my mind works at the moment. I believe that the world is a question of perspective and if i was to open this perspective i would be able to understand how i am everything. I believe that there are no limits to consciousness and most humans experience consciousness at the individual level.	Oneness, Subjectivity	An awareness of oneness with the world and the subjectivity of consciousness through perspective taking and shifting.
Psychedelics allowed me to see myself how I really was during a very hard period in my life, rather than how I was presenting myself to the world, and to me!	Insight, Authenticity	Psychedelics led to self-insight into (in)authenticity
I now view 'normal' as a socially constructed idea that only operates as a blanket term. Each individual person is unique and has their own will and desires, attempting to constrain these to fit into the mould of 'normal' only happens because people are often afraid of expressing themselves and find some comfort in becoming lost in a crowd of familiar faces.	Social constructivism, Difference acceptance	An awareness of the socially constructed nature of normality and an awareness and acceptance of people's individuality and uniqueness.
feeling that reality lies in the eyes of the beholder - it is not necessarily 'ultimate' - very subjective	Subjectivity	A sense of the subjective nature of reality.
I have a greater sense of empathy for others and forgiveness is something that comes very easily to me (this has maybe always been the case). I am interested in abolitionist thought and feel that psychedelic experiences have introduced me to a sense of connection with others that sits ever further at odds with the punitive and corrosive effects of the state.	Empathy, Interrelatedness	An increase in empathy aligned with psychedelic induced sense of interconnectedness that challenged their views on liberation and forgiveness.
I know see myself as part of a process of change, and one of many billions of lifeforms dependent on and contributing to the planet. I do not see a distinction between myself and other beings, I accept that all beings are in all others, and I think any artificial distinctions made by humans neglect the scale of the universe.	Boundlessness, Illusory boundaries, Interrelatedness	A sense of boundlessness, that boundaries are illusory, and beings are interconnected.
More inclined to pantheism (even though the word wasn't known to me then). I am certain that everything has a purpose and that we all are in this together. Nature has a plan.	Purpose and Values shifted, Animism, Interrelatedness	Attribution of consciousness to nature, understanding of interrelatedness and shared purpose.
There is no 'normal ' anymore. There's beliefs and choices, 'normal ' in my perception is a construct.	Rejecting Normal, Social constructivism	A rejection of the concept of normal and understanding of its constructed nature.
I am more in tune with the spirits of nature and regularly receive guidance and insights from source.	Nature connection, Attunement, Metaphysical resonance	Attunement with nature and a resonance with the metaphysical through that connection, experienced as guidance.

I have come into a place of realising an interconnectedness of everything, including human beings. This has created more of an 'us' attitude as opposed to 'us and them' and has made me understand people's pain as a source of what I perceive as maybe difficult behaviour. So, compassion has become and integrated part of how I perceive my world.	Compassion, Interrelatedness, Commonality	Awareness of interrelatedness with all, including other humans. An appreciation of commonality and understanding of pain behind difficult behaviour led to increased trait compassion.	**Increased prosociality and acceptance of difference (representing 29.41% of Graph)**
I would like to think I'm more accepting and open to diverse opinions, beliefs, personalities than before. Also, just much more interested in other people, their worlds, stories and experiences. Developed much more interest in helping people suffering from mental illness.	Difference Acceptance, Compassion	Increased openness, acceptance and interest in diversity of perspectives and experiences along with increased compassion and prosociality as a result.	
My world view was mechanistic and somewhat nihilist. I don't think I saw the inherent value in nature. I perceived matter as dead, rather than potentially experiencing entities in their own right.	Animism, Reductionism reduction	Increased attribution of consciousness to nature and a reduction of reductionist worldview.	
I try to trust my intuition more now in light of these experiences. I realised that we cut ourselves off from so much if we are constantly over-analysing and trying to rationalise things to ourselves constantly, it's not that this can't useful too, but I try to do it more situationally. I also walk around with much intuitive faith about spiritual aspects of existence and that the things revealed in those experiences are 'real' in some sense.	Trust and Faith in intuition, Metaphysical resonance	Increased trust in intuition and faith linked to metaphysical, spiritual aspects of existence.	
I am less compliant with outside demands; my intuition guides me through all decisions that I make. I connect to myself before taking any decisions and i would call that intuition.	Attunement to intuition, Alignment to self	Reduction in conformity, attunement to intuition as a guide	
I have become more spiritual, believe in an afterlife, in different forms of energies and so on- generally a move away from the rational scientific perspective (I am a scientist)	Reductionism reduction, Animism, Mortality	Increased recognition of different forms of energies and spirit that connect to a shift in perspective on mortality and move away from reductive scientific perspective.	
After the experience I had the insight that proper epistemology was crucial for navigating this confused world, so I became very interested in science & philosophy. Right after the experience I became a idealist (the philosophy of mind term), then gradually I noticed that I had been to easily convinced. Now I'm far more agnostic, but I see the importance of both religious and scientific worldviews. The most notable difference is that I'm now aware that the naive realist view I had before isn't plausible, and that my	Philosophical enquiry, Subjectivity, Agnosticism, Reductionism reduction	Increased interest in philosophical enquiry that led to agnosticism a shift away from reductionism with an understanding of the subjectivity that shapes perceptions of reality.	

mood/emotions/prior experiences shape how I perceive the world.			
I became a lot less reductive/physicalist in my beliefs and instead adopted a more mystical/consciousness first perspective. I became more open minded about a vast array of religious and spiritual traditions/practices/beliefs and many of their narratives made much more sense. I also made friends with death and see it as an exciting transformation.	Openmindedness, Mortality perception shift, Reductionism reduction	An increase in openmindedness about different beliefs and practices, shifting away from reductionist thinking and developing an acceptance of mortality.	
Psychedelics have shown me and given me more and more permission to be my true self. So, it's not like it gave me something wholly new that I never had before, it's more like it showed me something that I already had but didn't fully know was mine.	Authenticity, Insight	A sense of insight led to increased ability to connect to their authentic self	
On my first trip I felt my soul die and since then have become less fearful of death. My soul lifted from my body, and I went into a small white box-like room which I perceive to be death and the afterlife. I also believe I have met Mother Nature or some related female goddess on more recent trips, despite being atheist before taking psychoactive substances.	Mortality perception shift, Animism, Reductionism reduction	An experience of perceived soul death led to acceptance of mortality along with increased connection to animist beliefs and shift away from atheism.	
*The data was filtered using a Lift filter (Lift < 6.22), which filtered out any associations that were likely to have occurred by chance. A Leiden algorithm for cluster detection was used to identify thematic clusters in the graph, showing moderate modularity (Modularity = 0.228) with 3 clusters representing themes that show more connections with each other than with the rest of the graph.<br><br>**Note some quotes above include codes that belong to more than one cluster.			

B. Tables of identified themes, example quotes, frequency of coded theme and percentage of participant responses each theme was identified in.

Supplementary Table 1. Themes of the acute psychedelic experience

Theme	Example Quote	Freq	%
Emergence of insights and purpose	*Seeing things more clearly and a stronger sense of knowing how to move forward in terms of dealing with these issues*	21	23
Sense of oneness and connectedness	*It was just as the universe had opened up and I felt the connection to everything, everywhere.*	18	20
Suffering, death, challenging experience	*It was absolutely horrendous in terms of acute effects, and I felt I was dying,*	14	16
Experience of something greater than oneself	*I had drunk Ayahuasca, and I could clearly hear the medicine guiding me. I understood clearly what she was saying to me. Her voice was my voice, but it wasn't me or my higher self, it was something much greater/wiser*	12	13
Positive emotions (love, bliss, kindness, beauty)	*It was wonderfully reassuring to identify that the Universe and all things around us are inherently kind… The beauty of life itself is astonishing.*	11	12
Sense of profundity or higher reality	*It was as if a veil covering certain aspects of reality, which I wasn't aware of prior to this experience, was lifted.*	9	10
Religious or spiritual experience	*It felt like a very spiritual experience, as someone who is not particularly spiritual but open minded.*	7	8
Themes of nature	*A real connection with mother earth and plants.*	6	7
Expansion and wholeness of self	*…my sense of Self was expanded to include the wholeness of being. This interferes with the idea of a personal Self as experienced in normal consciousness, but it does NOT remove the sense of Self. I remained a Self throughout the experience with varying degrees of expansion/experiential limits.*	6	7
Questioning the nature of reality	*Confounding thoughts regarding the nature of the experience of being alive and aware.*	6	7

Post-experience epistemic changes

Supplementary Table 2. Themes of Intrapersonal changes: Changes in understanding of self

Theme	Example Quote	Freq	%

Greater self-insight	*I have had great insights into how I create my own obstacles at time [sic]. I can observe my inner narrative more objectively and have become increasingly aware of the generally negative nature of this*	35	42
Increased compassion	*yes, i* [sic] *am much more compassionate towards myself and more honest too*	22	24
Shift in purpose and values	*Yes, in that my actions and behaviour when using psychedelics have been, I think, generous and kind, which was sadly not always. So these experiences have helped me be - I hope - a better husband and father.*	21	23
Increased awareness of wholeness and complexity	*I see myself as more complex than I initially thought, i understand that there are many parts of myself that I do not always have access to, and that i often act in alignment with my conditioning rather than according to my true intentions and desires. I now understand much better the idea that my emotions do not jusitify my actions and that my emotions can be triggered by previous hurts and traumas.*	15	16
Increased authenticity	*I am more aware of what I want. I don't need to 'find myself', I understand the things that make me happy (long and short term) and surround myself with them.*	10	11
Wellbeing	*Because of my psychedelic experiences, I have the validation I need to keep going. Keep dreaming, creating, imagining, and adding onto this big beautiful thing we call a universe. I was suicidal for a long time until I woke up to my “difference” being extraordinary. I used to hate myself for being so different. Without psychedelics, I couldn’t have stayed the course. Without psychedelics, I would be lost in Southern provincialism.*	7	8
Increased awareness of oneness	*Loosened the idea that I'm a separate thing in an external world, more aligned with the religious idea that I am part of 'one thing doing everything'.*	7	8
Negative repercussions of change	*I’m still trying to understand what happened but I think that my ego may have been so inflated that when I did have the ego hit while on acid it hit me way harder…. it was such a horrible experience for me I’ve repressed it quite a bit.*	3	3

Supplementary Table 3. Themes of Interpersonal changes: Changes in understanding of others

**Theme**	***Example Quote***	**Freq**	**%**

Empathy	*Yes, I have a greater sense of empathy for others and forgiveness is something that comes very easily to me.*	25	27
Acceptance of difference	*Learning to adjust my own mapping of the world allowed me to understand that everyone has their own mapping and structures their own categories of evaluation often entirely different to my own.*	22	24
Awareness of commonality in human experience	*Understanding that everyone suffers and experiences fear*	21	23
Interrelatedness	*Very much changed. I have come into a place of realising an interconnectedness of everything , including human beings . This has created more of an 'us' attitude as opposed to 'us and them*	19	21
Increased compassion	*Yes, to be more compassionate and accommodating.*	16	18
Sense of sonder: recognizing wholeness of others	*Yes, I see everyone as an independent reality in and of itself*	9	10

Supplementary Table 4. Themes of Transpersonal changes: Changes in understanding of reality

**Theme**	**Example Quote**	**Freq**	**%**
Increased sense of animism	*I now know plants are much more alive than is taught in a science class. that they communicate with us in subtle ways and can be teachers for us*	26	29
Increased awareness of subjectivity of reality	*Yes, feeling that reality lies in the eyes of the beholder - it is not necessarily 'ultimate' - very subjective*	16	18
Shift in life purpose and values	*I think psychedelics have helped my clarify what's more and less important in my life.*	10	11
Interrelatedness	*The entire world became more connected. Although, my religious or spiritual beliefs remained somewhat intact - I am still an atheist without much of spirituality. The connectivity*	8	9

	part comes more from the inner workings of the Earth system and physical laws.		
Reductionism reduction	*I became a lot less reductive/physicalist in my beliefs and instead adopted a more mystical/consciousness first perspective.*	8	9
Increased religiosity	*Yes, I went from being an Atheist to a Christian. The experience pressed upon me that religion is something vital and necessary.*	8	9
Increase in philosophical enquiry	*led me to begin what has been a lifelong fascination for psychology, religion and philosophy.*	8	9
Increased openness and curiosity	*Returning to a more child-like perception of reality, reawakening my curiosity to explore its nature and boundaries (i.e., a more playful approach to understanding reality).*	7	8
Perception of mortality changed	*Yes - on my first trip I felt my soul die and since then have become less fearful of death.*	6	7
Increased agnosticism	*I'm more uncertain than I have ever been about any of my beliefs.*	5	6
Negative change	*It actually led me into a disastrous psychotic meltdown, not helped in any way by the adoption of eastern / new age / shamanistic belief systems that dissolved my hold on reality.*	2	2

Supplementary Table 5. Themes of changes in understanding what is 'normal'

Theme	Example Quote	Freq	%
Greater acceptance of difference and other perspectives	*Realising that it is all about perspective, and what is 'normal' for one it could be different to the other. There is no wrong or write* [sic]*, everything is how it should be.*	20	22
Seeing normality as a social construct	*Yes, definitely. I came to see during the experience that the "normal" is only a matter of our background (society beliefs, education etc) and not the really [sic] natural truths.*	19	21
Expanded concept of normality	*I'd say that the transformative experience vastly expanded what I thought was possible.*	18	20
Complexity Awareness	*increased awareness of the utterly astonishing complexity of the human brain. I have come to accept that "normal" is just statistics, merely a conceptual tool to help humans survive with the overabundance of information. It helped me appreciate that everyone has a different perception of normality and above all, that there is not a standard "trip" for everyone, meaning each individual will have a different experience with different drugs in different occasions.*	15	17

Metaphysical resonance	*Yes - my sense of the reality of God and sacredness has certainly increased*	10	11
Rejecting normal	*Absolutely. There is no 'normal ' anymore. There's beliefs and choices, 'normal ' in my perception is a construct.*	7	8
Nature connection	*Yes I have had a greater sense of interconnectedness when in nature, I now do more to prioritise being in nature and going to places which give me a sense of awe.*	6	7
Interrelatedness	*Yes, the entire world became more connected. From the interactions between Earth systems, like El Nino/La Nina, ocean or atmospheric circulations, to an individual level between people… The 'new' normal became the connection between the nature, people and the universe, where respect for individual beings and their place among these connections become a default thinking rather than an afterthought or conclusion.*	4	4
Negative change	*Yes, I am now very paranoid and normal things seem to induce a fear response*	2	2

Supplementary Table 6. Themes of Changes in intuition

**Theme**	**Example Quote**	**Freq**	**%**
Greater guidance and attunement to intuition	*I am less inclined to do things that do not ring true to who I am. I am less compliant with outside demands, my intuition guides me through all decisions that I make. I connect to myself before taking any decisions and i would call that intuition.”*	19	21
Greater trust and faith in intuition	*Yes very much so. I felt like I have learned to trust more in life and its ways. Thus, I also was able to trust my intuition more. I was more freed from my past self and the boundaries given to me by society.*	16	18
Shift in purpose and values	*I observed a massive shift in what mattered to me e.g. no longer caring for material things and stressing less over things I can't control.*	15	17
Interrelatedness	*The way in which I raise my children transformed markedly based on the psychedelic experience. It is beautiful to be in a family where each person is thankful for all others, and each one interested in participating together.*	13	14
Metaphysical resonance	*A greater intuition that there is *something* to spirituality and there *is* something deeply sacred to everything around us, which has evolved into a wider acceptance of God.*	12	13

Openness	*I think it's shifted something and made it easier to feel open and mindful about things.*	9	10
Greater alignment with self	*I am now more aware of everything, I would say conscious, especially to what my body feels, therefore I am more connected to my feelings.*	8	9
Complexity awareness	*It also made me better at comprehending complex ideas, I think it genuinely made me develop a more nuanced understanding of how abstract ideas like economics and people work as I was thinking more deeply about them in a less biased way.*	7	8
Nature connection	*Yes completely… I feel much more in tune with nature and feel the need to sometimes take a break from life and spend time in nature which helps me a lot. I feel an extreme sense of connectedness with nature.*	6	7
Empathy	*I feel I can better empathise and understand both other peoples emotions and also my own. understanding my own experiences and emotions has had a massive impact on my life.*	5	6
Negative Change	*I have since been left in a state of psychosis, and have had my life ruined because of this substance, I wish I had never touched them*	4	4

Supplementary Table 7. Themes of changes in boundaries between self and world

**Theme**	**Example Quote**	**Freq**	**%**
Interrelatedness	*I realized [sic] that everything has its place in a grand scheme of existing. We all belong together, and life is one big force connecting us and our paths*	27	30
Malleability	*I see the boundaries as a lot more malleable and interchangeable. In many of my experiences I took on and became aware of vastly different states of consciousness.*	11	12
Dismissing boundaries as illusory	*I believe that the boundaries we see between ourselves, and the rest of the world are a temporary illusion.*	10	11
Boundlessness	*Borders and boundaries removed.*	9	10

Oneness	*Whilst I still maintain a separation in the experiential sense, I feel and know that I am in many ways metaphysically Indistinct*	9	10
Increased disconnection	*I am now more closed minded and feel more isolated.*	2	2

C. Participants who evaluated their transformative experiences as overall negative

Four participants self-identified their significant psychedelic experiences as negative. Two of these participants had taken psilocybin, one reporting subsequent hallucinogen persisting perception disorder (HPPD) with worsening palinopsia more than a year later, and another describing persistent psychosis, paranoia, depression, and suicidality following an initially positive experience. Two of the participants transformative experiences involved LSD, one during a prolonged period of heavy use, which resulted in psychosis, paranoia and flashbacks, while the other was during a single adolescent experience that led to enduring trauma but eventual partial reframing through psychotherapy.

P. No	Substance	Age at time of experience	Time since psychedelic experience	Setting	Main persisting negative outcomes
1	Psilocybin	29	2 years	Home, with twin brother	Psychosis, Paranoia, suicidality
2	Psilocybin	-	2 years	Home, alone	HPPD/Palinopsia
3	LSD (heavy use)	16	40 years	Various, isolated	Psychosis, Flashbacks
4	LSD	18	34 years	Party, Friends	Traumatic experience

These four participants ranged from age 16 to 29 at their first transformative experience and from 6 months to over 40 years since their last psychedelic use. Contexts were typically non-clinical and unstructured. Social environments were mixed, with one participant alone, another with his twin brother, and others in social gatherings.

These participants described lasting disturbances in self-perception and reality testing. One wrote, “I have since been left in a state of psychosis, and have had my life ruined because of this substance, I wish I had never touched them”. Another reflected, “At the most extreme

points in the psychedelic / psychotic experience I saw everything as a creation of my mind. I was the only being who existed and everything was effectively my imagination and not actually real.” Others reported difficulty processing or integrating the event: “A part of me still feels a bit stuck in that bad trip experience- it's hard to explain that but it's taken a long time to work out the trauma and sometimes it feels like a part of me got stuck there.”

These narratives revealed a collapse of meaning making rather than its expansion, in contrast to participants with positive experiences who emphasized unity, gratitude, and insight. Themes of loss of control, existential threat, and social disconnection appear in their responses. One participant described negative reactions after talking about their experience with “my family, friends, and work colleagues; this resulted in being fired from work.”
